# A Reciprocal Model of Face Recognition and Autistic Traits: Evidence from an Individual Differences Perspective

**DOI:** 10.1371/journal.pone.0094013

**Published:** 2014-05-22

**Authors:** Drew W. R. Halliday, Stuart W. S. MacDonald, Suzanne K. Sherf, James W. Tanaka

**Affiliations:** 1 University of Victoria, Victoria, British Columbia, Canada; 2 The Pennsylvania State University, State College, Pennsylvania, United States of America; 3 The Centre for Autism Research, Technology and Education, Victoria, British Columbia, Canada; Lyon Neuroscience Research Center, France

## Abstract

Although not a core symptom of the disorder, individuals with autism often exhibit selective impairments in their face processing abilities. Importantly, the reciprocal connection between autistic traits and face perception has rarely been examined within the typically developing population. In this study, university participants from the social sciences, physical sciences, and humanities completed a battery of measures that assessed face, object and emotion recognition abilities, general perceptual-cognitive style, and sub-clinical autistic traits (the Autism Quotient (AQ)). We employed separate hierarchical multiple regression analyses to evaluate which factors could predict face recognition scores and AQ scores. Gender, object recognition performance, and AQ scores predicted face recognition behaviour. Specifically, males, individuals with more autistic traits, and those with lower object recognition scores performed more poorly on the face recognition test. Conversely, university major, gender and face recognition performance reliably predicted AQ scores. Science majors, males, and individuals with poor face recognition skills showed more autistic-like traits. These results suggest that the broader autism phenotype is associated with lower face recognition abilities, even among typically developing individuals.

## Introduction

While repetitive behaviours, delayed language and impaired social function are the hallmarks of autism, many individuals on the autism spectrum experience problems recognizing faces [1]–[2] and interpreting facial expressions [3]. Despite the well-established link between autism and face recognition [2], [4]–[7], it is less clear whether impaired face recognition is the consequence of, or a contributor to, the autistic condition. On one hand, a reluctance to socially engage with others and an aversion to making eye contact will invariably lead to less perceptual experience with faces, which in turn, could result in impaired face processing ability [4]. On the other hand, if impoverished face recognition skills are systemic to the autistic condition, a compromised face processing system might interfere with everyday social interactions, (e.g., social confusion and miscommunication) and further exacerbate the autistic condition.

In this study, we employ a hierarchical multiple regression method to examine the reciprocal relation between face recognition and autistic traits in a sub-clinical population. Our main findings are that whereas autistic-like tendencies reliably predict poor face recognition performance, the converse is also true, that impaired face processing predicts autistic-like tendencies.

Autism spectrum disorder (ASD) is characterized by deficits in communication and social interactions, displays of repetitive stereotypic behaviours, and expressions of restricted interests [8]. By definition, autism is a spectrum disorder with individuals presenting a range of symptoms that are classified as mild to extreme in their severity. As such, the autism continuum extends to people who may lie outside the formal autism diagnosis, but who nevertheless display autistic-like behaviours, [9]–[11]. The Autism Quotient (AQ) was developed as a self-assessment questionnaire, designed to measure a person's degree of autistic traits [11]. Participants rate their level of agreement on a four-point scale to statements indicative of ASD characteristics (e.g., When I talk, it isn't always easy for others to get a word in edgeways). The AQ measure has been used to identify group differences in the Broader Autism Phenotype [12]. For example, studies have shown that undergraduate science majors score significantly higher on the AQ measure than non-science majors [11], [13]. By adopting an individual differences approach, our goal is to study a potential endophenotype of autism in the larger, subclinical ASD population, to yield important insights into the roots of the disorder.

In the autism literature, a clear link has been established between the autistic condition and impaired face processing abilities. Compared to their typically developing (TD) peers, individuals with ASD show less interest in faces [14]–[16] and perform worse on measures of face memory [e.g.], [ 2], [7,17]. Brain regions that are differentially activated by faces (i.e., fusiform gyrus) show reduced activation in groups of adults [4], [18] and adolescents [19] with ASD. However, the level of fusiform activity exhibited by persons with ASD may depend on whether they are attending to the eyes of the face [20], viewing a familiar or unfamiliar face [21] or are socially anxious [22]. Within the TD population, individuals who report high degrees of autistic characteristics also exhibit functional and structural brain abnormalities in neural regions involved in social face processing [23]. Studies employing event-related-potential (ERP) methods indicate that the brain response to faces is delayed in individuals with ASD relative to TD control participants [24].

Although persons with autism display selective deficits in their face processing skills, it is less clear whether individuals with face deficits present autistic-like tendencies. Developmental prosopagnosia is a selective deficit in face recognition skills due to unspecified, congenital origins. Not surprisingly, persons with a life-long impairment in their ability to recognize familiar faces experience a great deal of anxiety that forces them to avoid social situations [25]. However, growing up with a severe face recognition deficit does not necessarily lead to the development of autistic-like behaviours. In one study, a sample of individuals with developmental prosopagnosia completed the AQ and other social cognition measures [26]. The questionnaire results indicated that these individuals were not prone to autistic behaviours nor were they impaired in their social interactions. As with ASD, individuals with prosopagnosia are impaired in their face recognition abilities, however unlike ASD individuals, they are motivated to be socially engaged and to develop compensatory strategies for person recognition (e.g., identification through clothing, voice or gait cues). Although face deficits do not predict autistic tendencies, it is possible that people with autistic tendencies are susceptible to face processing difficulties that further exacerbate the autistic condition.

In the current study, we explore the potential reciprocal relationship between face recognition abilities and the autistic phenotype in a large, non-clinical sample of university students. Related studies have shown that face recognition skills are correlated with measures of social cognition in the TD population. For example, individuals who perform worse on measures of face memory report low degrees of empathy [27] and extraversion [28]. Although these studies controlled for visuospatial and object recognition abilities, they used group differences to compare the high and low ends of their respective distributions (i.e., empathy or extraversion) to do so. By examining group differences (e.g., comparing face and object recognition abilities in individuals scoring high vs. low on a proxy of social cognition) rather than individual differences, the shared variance between face and object recognition is not properly accounted for (for a discussion, see [29]). Examining individual differences facilitates not only investigating the unique contributions of predictor variables (e.g., face and object recognition abilities), it also examines variance within the entire distribution of scores, rather than the high and low ends exclusively. Here, we employ a hierarchical multiple regression model to isolate the relationship between autistic traits and facial and emotion recognition abilities, while controlling for general visuospatial and object recognition abilities. We predict that a person's degree of self-reported autistic traits (i.e., their AQ score) will be uniquely associated with their ability to recognize facial identity and facial expression. Accordingly, individuals who report higher degrees of autistic traits will show a selective deficit in facial and emotion recognition.

## Methods

### Ethics Statement

The institutional review boards at both the University of Victoria and Carnegie Mellon University approved this study.

### Participants

131 participants (85 females) aged 18–30 years (*m* = 20.4 years, *SD* = 2.6) were recruited to participate in this study. 26 participants from Carnegie Mellon University (20 males) were recruited from the Computer Science and Engineering departments. 105 participants from the University of Victoria (79 females) were recruited from various introductory Psychology classes. In total, participants identified 31 different majors.

### Procedures

Participants gave written informed consent and were presented with the battery of measures in the following order: Immediate Memory Face task, Immediate Memory Bird task, Emotion Recognition task, Embedded Figures Test, and Autism Quotient (AQ). Participants completed the experiment individually in a quiet room, on computers equipped with Intel Pentium 4 processors and 15-inch Sony Trinitron E240 or LG Flatron F700P monitors, set at a screen resolution of 1024 by 768 pixels. Participants were compensated with bonus course credit or money ($10), and were debriefed immediately after completing the experiment.

#### Immediate Memory (IM) Face Task

This task measured participants' short-term memory for novel faces by requiring them to match identity, and was adapted from the original *Let's Face It!* skills battery [2]. A study face was shown in frontal view for 1,000 msec, followed by a noise mask for 500 msec, and finally three probe faces at the same 3/4 orientation for 3,000 msec. Participants chose the target face corresponding to the study face based on identity from the three alternative choices. Face stimuli included grey-scaled images from the Joe Stein Face Set with external features removed. Images subtended 5.28°×7.17° of visual angle in the horizontal and vertical dimensions, respectively. There were 30 trials in total presented in fixed sequential order.

#### Immediate Memory (IM) Bird Task

This task measured participants' short-term memory for birds, and served as a control measure for the IM Face task. As in the face identity task, a study image was shown for 1,000 msec, followed by a noise mask for 500 msec, and finally three probe images for 3,000 msec. The probe images were all from the same genus, with the correct image corresponding to the exact same species of bird as the study image. Sparrows, Warblers, Orioles, Tanagers, Blackbirds, Buntings and Flycatchers comprised the stimulus set. Images were grey-scaled and subtended visual angles of 5.65°×7.05° in the horizontal and vertical dimensions, respectively. There were 30 trials in total presented in fixed sequential order.

#### Emotion Recognition Task

This task measured participants' ability to recognize six basic emotions across different identities. As in the previous tasks, a study face was shown for 1,000 msec, followed by a noise mask for 500 msec, and finally three probe faces in frontal view for 3,000 msec, one of which depicted the same emotion as the study face. Faces were chosen from the Karolinska Face Set [30] and expressed the following emotions: sadness; anger; fear; happiness; disgust and surprise. Images were grey-scaled, contained external features, and subtended visual angles of 5.28°×7.17° in the horizontal and vertical dimensions, respectively. There were 30 trials in total presented in fixed sequential order.

#### Embedded Figures Test

This task involved disembedding simple shapes from complex visual scenes and was adapted from the original pencil-paper version [31]. This task served to measure participants' global ability for visuospatial perception; our ability to process information about where objects are in space. Participants viewed each scene separately and completed as many of the 16 trials as possible within a 12-minute time period by choosing from five basic shapes, the one that was hidden within the complex figure.

#### Autism Quotient

The Autism Quotient (AQ) is a 50-item self-report questionnaire based on five subscales (communication, social interaction, imagination, attention to detail, and attention switching) relating to the triad of impairments characterizing ASD [11]. It is designed for use with people of normal intelligence to quantify their degree of autistic traits. Participants rated each statement as either ‘definitely agree,’ ‘slightly agree,’ ‘slightly disagree,’ or ‘definitely disagree.’

#### Analyses

To test our primary hypotheses, we ran an initial correlation to examine the relationship between autistic traits (AQ) face identity recognition (IM Face), object recognition (IM Bird) emotion recognition, Embedded Figures, gender and major. Then, we employed hierarchical multiple regression in order to test the relative predictive contribution of variables entered in a blocked-entry order. Two hierarchical regression models were assessed, differing in terms of the dependent measure (AQ scores or IM Face scores). Given our strong, a priori hypotheses, we evoked one-tailed p-values for our regression analyses.

For the first block, gender and major were entered consistent with their robust associations to AQ scores reported in previous studies [e.g., 11]. The final stage of each model was reserved for AQ or identity recognition, in order to employ a conservative test of whether either variable remained a unique predictor after partialling out variance linked to the other theoretically motivated variables. We maintained a consistent order of variable entry across models to facilitate direct comparison, and report one-tailed p values in accordance with our a priori and directional hypotheses. Prior to these analyses, we examined skewness and kurtosis values (see Table 1) for each univariate distribution, as well as the scatterplots for each bivariate distribution. All data were univariate normal based on conventional standards (i.e., no values were greater than ±3.27). There were no multivariate outliers, based on visual inspection of residual plots, as well as Mahalanobis distances.

**Table 1:** Descriptive Characteristics.

	Mean	SD	Range	Skewness	Kurtosis	Cronbach's α
IM Face	0.68	0.11	.43–.93	−1.07	−1.16	.53*
IM Bird	0.63	0.09	.37–.80	−1.98	0.53	.62*
Emo Rec	0.68	0.09	.37–.93	−1.63	1.32	.45*
EFT	−2.37	6.70	−16–15	1.97	−0.79	
AQ	55.38	11.35	23–90	−0.14	1.34	

*Based on previous piloting.

## Results

Seven participants did not declare a major, and were not included in the analyses. Scores on the IM Face (*M* = .68, *SD* = .11) and the IM Bird (*M* = .63, *SD* = .09) measures were of comparable difficulty, and produced distributions with similar variability. Scores on the Embedded Figures Test were computed by subtracting incorrect responses from correct ones across trials attempted (*M* = −2.37, *SD* = 6.70), yielding a potential range of −16–16, where high scores correspond to superior featural processing. Table 1 summarizes the descriptive characteristics of each measure. Scores on the Autism Quotient ranged from 23–90 (*M* = 55.38, *SD* = 11.35). In keeping with recent studies that have used the AQ [e.g.], [ 32–33] we used a 4-point scoring system rather than the original 2-point system in order to increase variability amongst scores to better examine individual differences. With the 2-point scoring system, a score of 32 is suggested to correspond with clinical cutoff [11]; with a 4 point scoring system, this translates to a score of 96. University majors were dichotomized into Science and non-Science majors, according to the criteria used by Baron-Cohen et al. [11].

### Correlations among all measures

**Figure 1:**
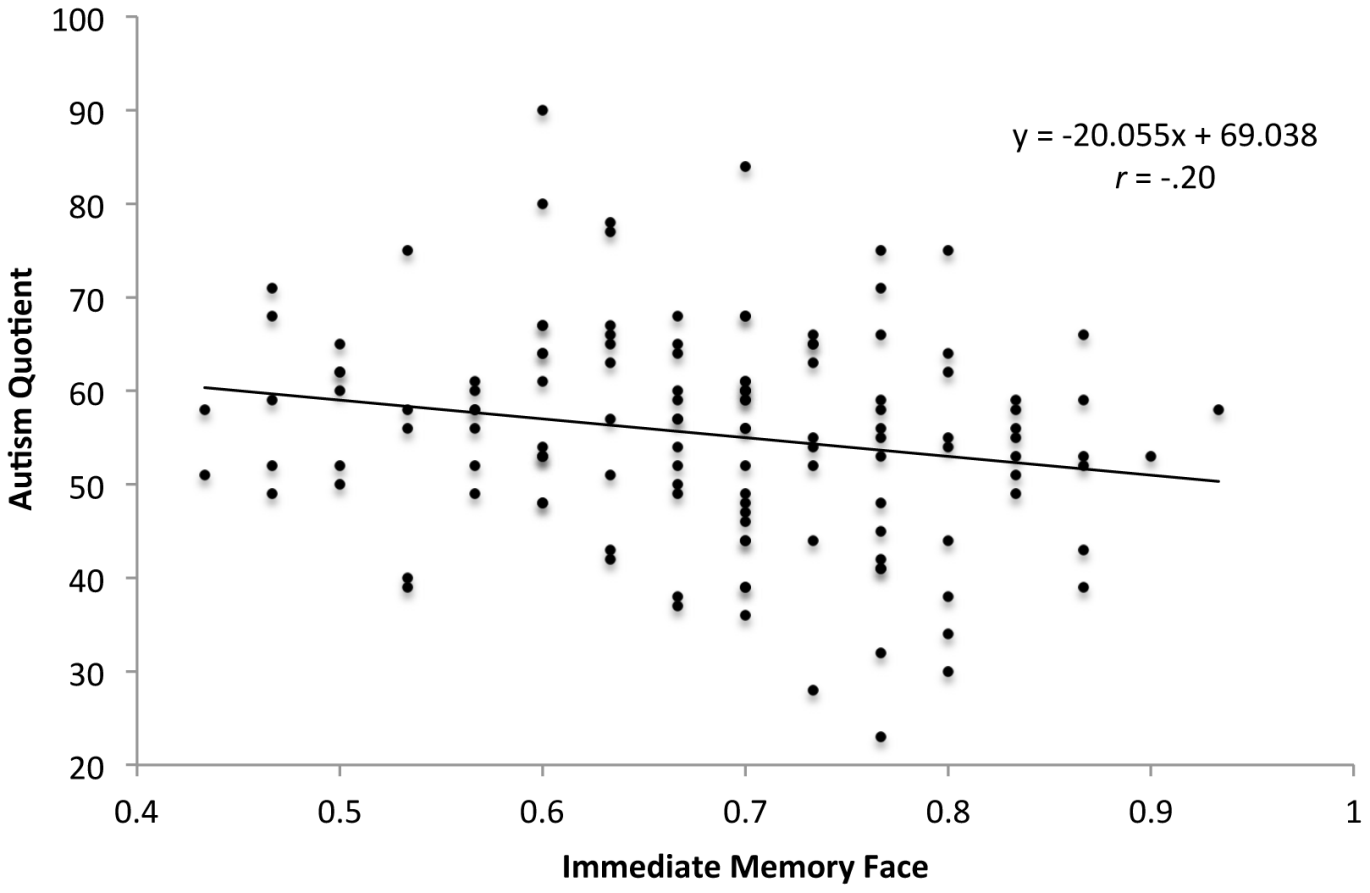
Autism Quotient scores as a function of Immediate Memory Face scores.

A Pearson product-moment correlation coefficient was computed to assess the relationship between scores on the Autism Quotient (AQ), and scores on the other four measures. Table 2 displays the correlations between all measures in the study. There was a significant negative correlation between AQ scores and scores on the IM Face, *r* = −.20, *p* = .02; a scatterplot summarizes these results (see Figure 1). The correlations between AQ scores, and scores on the IM bird, Emotion Recognition and Embedded Figures tasks were not significant (*p*>.05). These results suggest that there is a specific relationship between the magnitude of autistic traits and face recognition behaviour, and not a more general relationship between visual processing abilities and autistic traits. The correlations between AQ scores and gender (*r* = −.22, *p* = .01), and AQ scores and university major (*r* = −.27, *p* = .002) were also significant. Given the lack of relation with AQ scores, the Emotion Recognition and EFT measures were not included in subsequent analyses. The IM Bird task was retained in subsequent analyses as some studies have found superior object recognition abilities in individuals with ASD [2]. Moreover, the IM Bird and Face tasks are identical in their presentation of stimuli, which affords a comparison of the relationship between autistic traits, and face and object recognition abilities.

**Table 2:** Correlations between all measures.

	Major	IM Face	IM Bird	Emo Rec	EFT	AQ
Gender	*r* = .43**	*r* = .17*	*r* = .10	*r* = .24**	*r* = −.08	*r* = −.22**
Major		*r* = .07	*r* = −.07	*r* = .06	*r* = −.01	*r* = −.27**
IM Face			*r* = .22**	*r* = .19*	*r = −.08*	*r* = −.20*
IM Bird				*r* = .39**	*r* = .28**	*r* = −.10
Emo Rec					*r* = .15	*r* = −.03
EFT						*r* = −.05

*p<.05.

**p<.01.

### Predicting Face Recognition Performance - Multiple Regression

A hierarchical multiple regression was performed modelling IM Face scores as a function of gender, major, IM bird scores, and AQ scores. Gender and major were entered in the first block of the model, followed by IM bird scores in the second stage, and AQ scores in the final stage. AQ was placed in the final block to determine whether it uniquely predicted IM Face scores independent of the other variables.

**Figure 2:**
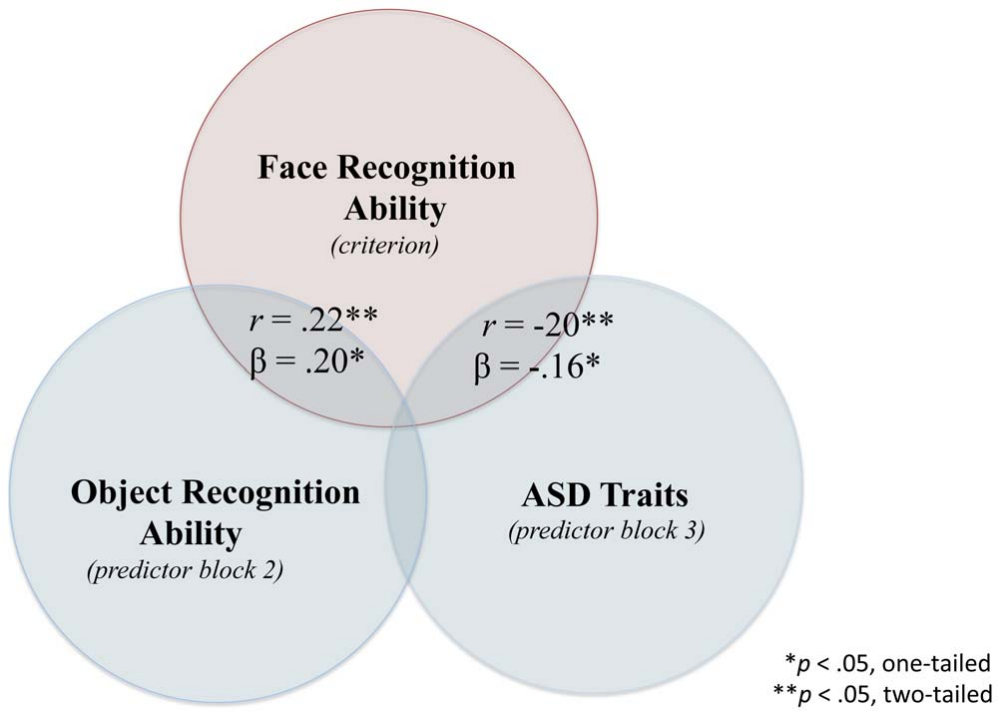
Venn representation of hierarchical regression analysis with face recognition ability (IM Face) as the criterion variable showing shared and unique variance with predictor variables. The variables presented are “selective” (i.e., gender and major are omitted from the diagram), however their effects are controlled for in the betas reported. Selected predictor variables include object recognition (IM Bird) and ASD traits (AQ) with β values reported at the stage the variable was entered. The overlap between predictors is not represented to scale and is for illustrative purposes.

Table 3 displays the unstandardized regression coefficients, intercepts, βs (standardized coefficient), SE B, semi-r and *p* values for each stage of the hierarchical regression model. The overall regression model including all 4 predictors simultaneously was significant, *F*(4,119) = 2.88, *p* = .03, *R^2^* = .09. In the final model with all variables entered, block 2 object recognition (β = .18, *p* = .03, one-tailed) and block 3 AQ (β = −.16, *p* = .04, one-tailed) significantly predicted face recognition scores. Notably, AQ emerged as significant, despite being entered in the final stage of the regression analysis. Figure 2 depicts the shared and unique variance between selected predictor variables and IM Face scores.

**Table 3:** Hierarchical regression on face recognition scores as a function of university major, gender, bird recognition and Autism Quotient.

Variables	B	SE B	β	semi-r	*p*
Stage 1					
Intercept	0.66	0.03			
Gender	0.04	0.02	0.17	0.15	0.05
Major	−0.00	0.02	−0.01	−0.01	0.40
Stage 2					
IM Bird	0.25	0.12	0.20	0.20	0.02
Stage 3					
AQ	−0.00	0.00	−0.16	−0.16	0.04

* *p* values are one-tailed.

### Predicting AQ scores – Multiple Regression

**Figure 3:**
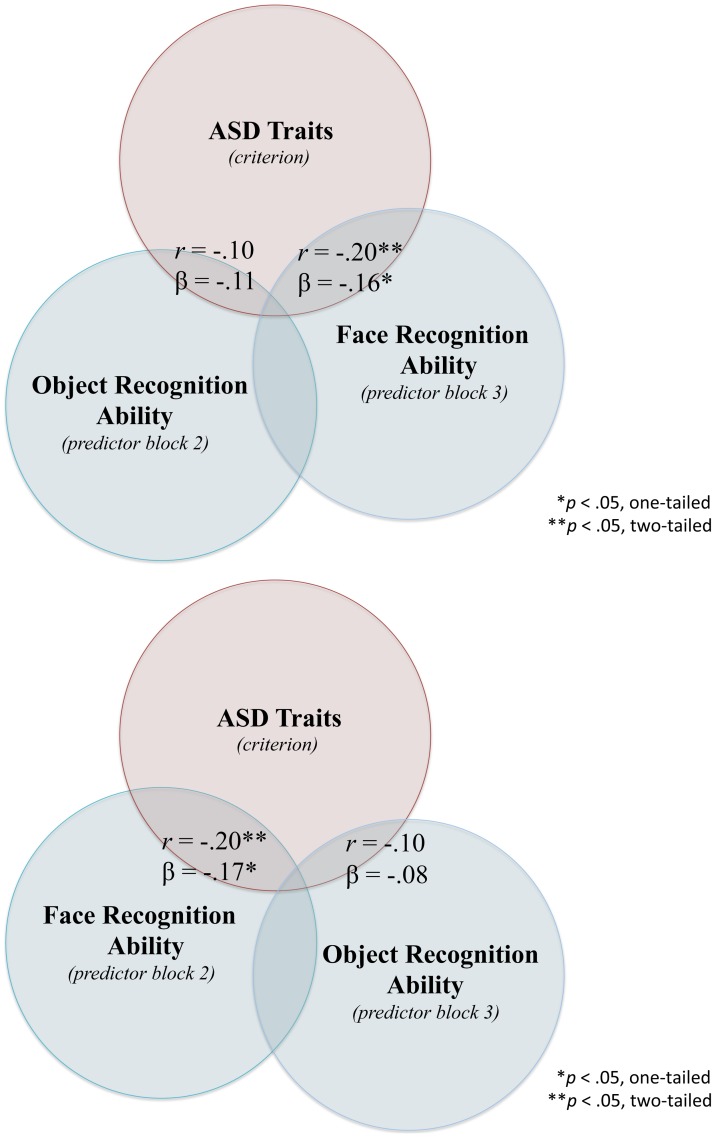
Venn representation of hierarchical regression analysis with Autism Quotient scores as the criterion variable showing shared and unique variance with predictor variables. The variables presented are “selective” (i.e., gender and major are omitted from the diagram), however their effects are controlled for in the betas reported. (*a*) Selected predictor variables include stage 2 face recognition (IM Face) and stage 3 object recognition (IM Bird) or (*b*) stage 2 object recognition (IM Bird) and stage 3 face recognition (IM Face); β values reported at the stage the variable was entered. The overlap between predictors is not represented to scale and is for illustrative purposes.

A separate hierarchical multiple regression was performed modelling AQ scores as a function of gender, major, IM bird scores, and IM Face scores. Whilst zero-order bidirectional associations were established in the initial correlation analysis, in the hierarchical analysis we sought to examine the partial associations between key predictors of interest and AQ scores. In order to assess the unique predictive power of face recognition (stage 2) over object (bird) recognition (stage 3), as well as the converse, we performed two regression analyses that switched the entry order of stages 2 and 3. Table 4 displays the unstandardized regression coefficients, intercepts, βs (standardized coefficient), SE B, semi-r and *p* values for each stage of the hierarchical regression model. As expected, when entered in stage 1, major significantly predicted individual differences in AQ scores, with a near significant association with gender. Entering IM Face in stage 2 significantly predicted AQ scores after accounting for the stage 1 measures. This remained true even when IM Face was entered last in stage 3, following gender, major, and IM bird. In contrast, a notable dissociation was observed for IM bird, which failed to uniquely predict AQ scores in stage 2 or 3 (i.e., before or after IM Face). The overall regression model including all 4 predictors simultaneously was significant *F*(4,119) = 4.33, *p* = .003, *R^2^* = .13. In this final model, block 1 university major (β = −.22, *p* = .02, one-tailed) and block 3 face recognition (β = −.16, *p* = .04, one-tailed) uniquely predicted self-reported autistic traits. Figure 3 depicts the relationships between selected predictor variables and AQ scores.

**Table 4:** Hierarchical regression on Autism Quotient scores as a function of university major, gender, face recognition, and bird recognition.

Variables	B	SE B	β	semi-r	*p*
Stage 1					
Intercept	65.27	3.35			
Gender	−3.61	2.35	−0.15	−0.14	0.06
Major	−4.75	2.28	−0.20	−0.19	0.02
Stage 2					
IM Face	−17.69	8.93	−0.17	−0.18	0.03
Stage 3					
IM Bird	−10.38	11.68	−0.08	−0.08	0.20
Stage 2					
IM Bird	−10.38	11.67	−0.08	−0.08	0.18
Stage 3					
IM Face	−16.11	9.11	−0.16	−0.16	0.04

The order of the final two variables in the model (IM Bird and IM Face) was altered in two separate analyses to show that even when IM Face was entered last, it emerged as a significant predictor.

* *p* values are one-tailed.

### Comparing AQ scores across Majors

To compare AQ scores across university major, we grouped majors into Sciences (*n* = 52), and non-Science majors (i.e., Social Sciences and Humanities; *n* = 72), and found significant differences between the two groups. Science majors (*m* = 59.13, *SD* = 11.12) scored higher on the AQ than non-Science majors (*m* = 52.76, *SD* = 11.18), *t*(2,122) = 3.14, *p* = .002. This finding is consistent with other studies [11], [13], [34], where Science majors have reported more autistic traits than non-Science majors.

## Discussion

In this study, we investigated how sub-clinical autistic traits and face recognition in the TD population are related to the demographic variables of gender and university major as well as to cognitive abilities involved in visual perception, object and emotion recognition. The pairwise correlations showed reliable associations between self-reported autistic traits with gender and university major. These results are consistent with previous findings in which autism has been linked to gender [35]–[36], university major [11] and face processing skill [1]–[2]. Face recognition ability correlated with gender, object recognition, emotion recognition and AQ.

To better understand the unique contributions of the factors to face recognition and AQ, we performed two hierarchical regression analyses. In Model 1, we investigated whether gender, major, IM Bird recognition and AQ measures reliably predicted IM Face recognition performance. In Stage 1, gender and university major were simultaneously entered into the model (see Table 3). The analysis showed that gender reliably predicted face recognition abilities where women tended to perform better on the IM Face recognition measure than men, which is in keeping with previous findings [37]–[38]. In Stage 2 of the model, the IM Bird recognition measure reliably predicted IM Face recognition indicating that object recognition uniquely contributes to face recognition ability [39]–[40]. In Stage 3 of the model, the AQ measure reliably predicted scores on the IM Face measure. This result indicates that face recognition deficits predict autistic tendencies in a sub-clinical population similar to the face processing deficits observed in individuals who are formally diagnosed with autism [1]–[2].

In a second model, we investigated whether gender, major, IM Bird recognition and IM Face recognition measures reliably predicted AQ performance. In Stage 1, gender and university major were simultaneously entered as the first factors into the model (see Table 4). Consistent with previous results [11], [13], [34], the analysis revealed that major was significantly predictive of AQ score such that students who majored in the sciences (Engineering, Computer Sciences and Mathematics) tended to score higher on the AQ scale than non-Science students. In Stage 2, two versions of the model were tested in which either the object recognition measure (IM Bird) was entered in Stage 2 and the face recognition measure (IM Face) (Model 2A) in Stage 3 or the converse order in which face recognition measure was entered in Stage 2 and the object recognition measure in Stage 3 (Model 2B). In Model 2A, the analysis showed that the IM Face measure reliably predicted AQ scores, indicating that face recognition abilities uniquely contributed to autistic characteristics. The IM Bird measure did not capture any additional variance when it was entered as a predictor in Stage 3 of the model. When the order of entry was reversed in Model 2B, the IM Bird measure still failed to predict AQ scores in Stage 2 whereas the IM Face measure continued to be a reliable predictor of AQ when entered last in Stage 3. Although the shared variance between face and object recognition performance is reliable (*r* = .22, Table 2), only the face recognition measure uniquely predicted autistic characteristics as measured by the AQ scale.

Dawson et al. [41] note that there is a reciprocal relationship between impaired face perception and impaired social cognition in young individuals with ASD. They propose that neither faces nor social interactions elicit the same reward value that they do for typically developing children. The implications of this are twofold and not necessarily mutually exclusive. Firstly, a primary perceptual deficit such as impaired facial recognition can have broader implications for aspects of social cognition that rely on the face (e.g., engaging in joint attention, recognizing emotions). Conversely, facial recognition deficits may be a byproduct of a lack of social motivation (i.e., faces are not tagged with positive affect because the individual is not interested in social stimuli). Similarly, Schultz et al. [4] found that the level of fusiform gyrus activation correlated negatively with the social cognition subscale of Autism Diagnostic Observation Schedule. The developmental trajectories of face perception and social cognition appear to be intimately related, and they likely influence one another over time. Notably, gender appears to affect the direction of the relationship between face identity coding and social interactions, with males but not females showing a positive correlation [33].

Our findings show that a person's degree of autistic traits in the typically developing population can be predicted by their performance on an identity recognition measure, one with relatively few trials. This measure is sensitive to individual differences in face recognition, and provides an implicit insight to social cognition, which is a limiting factor for individuals with the autism condition. We propose that it is equally plausible for the relationship between impaired face perception and core ASD traits to work both ways. On one hand, our findings suggest that the autism condition can lead to a general disinterest or aversion to social stimuli, such as faces, and produce a decline in face recognition skill (see Figure 2). On the other hand, our results suggest that it is equally plausible that impaired face perception can exacerbate core ASD symptoms pertaining to the social and communicative aspects of the condition (see Figures 3a and 3b). The implications of these findings are important for developing intervention programs that focus on face training and creating reward systems that emphasize the importance of faces from an early age [42]. These findings provide a tractable treatment strategy for ameliorating the symptoms of autism through face training, which in turn, may improve the quality of social interactions for individuals with ASD. Effectively, intervention programs targeting the recognition of faces may not simply be targeting a symptom of ASD, but may instead be targeting a more fundamental deficit of the disorder.

Although we make no claims about the direction of causality, our results show that the relationship between impaired face perception and core ASD traits can work both ways, and suggest that the relationship is dynamic and mutually reinforcing. We note however, that additional research (e.g., using large-sample, longitudinal designs with lead-lag models to examine differences in the onset of deficits) will ultimately be required to address the circular nature of the relationship. We propose that a primary deficit in social interactions is possibly compounded by an impaired ability to recognize the human face, as it is a highly social stimulus from which we infer a great deal of information that contributes to our social functioning. Facial recognition deficits are not a causal factor to developing ASD; however, we suggest that they may exacerbate the autism condition.
