# Correction: Correction: A Reciprocal Model of Face Recognition and Autistic Traits: Evidence from an Individual Differences Perspective

**DOI:** 10.1371/journal.pone.0133092

**Published:** 2015-07-13

**Authors:** The PLOS ONE Staff

The third author’s name is spelled incorrectly in the correction published on August 11, 2014. The correct name is: K. Suzanne Scherf. The correct citation is: Halliday DWR, MacDonald SWS, Scherf KS, Tanaka JW (2014) A Reciprocal Model of Face Recognition and Autistic Traits: Evidence from an Individual Differences Perspective. PLoS ONE 9(5): e94013. doi:10.1371/journal.pone.0094013. The publisher apologizes for the error.
